# Production of gamma-aminobutyric acid by *Lactobacillus brevis *NCL912 using fed-batch fermentation

**DOI:** 10.1186/1475-2859-9-85

**Published:** 2010-11-12

**Authors:** Haixing Li, Ting Qiu, Guidong Huang, Yusheng Cao

**Affiliations:** 1 State Key Laboratory of Food Science and Technology, Nanchang University, Nanchang 330047, PR China; 2 Sino-German Joint Research Institute, Nanchang University, Nanchang 330047, PR China

## Abstract

**Background:**

Gamma-aminobutyric acid is a major inhibitory neurotransmitter in mammalian brains, and has several well-known physiological functions. Lactic acid bacteria possess special physiological activities and are generally regarded as safe. Therefore, using lactic acid bacteria as cell factories for gamma-aminobutyric acid production is a fascinating project and opens up a vast range of prospects for making use of GABA and LAB. We previously screened a high GABA-producer *Lactobacillus brevis *NCL912 and optimized its fermentation medium composition. The results indicated that the strain showed potential in large-scale fermentation for the production of gamma-aminobutyric acid. To increase the yielding of GABA, further study on the fermentation process is needed before the industrial application in the future. In this article we investigated the impacts of pyridoxal-5'-phosphate, pH, temperature and initial glutamate concentration on gamma-aminobutyric acid production by *Lactobacillus brevis *NCL912 in flask cultures. According to the data obtained in the above, a simple and effective fed-batch fermentation method was developed to highly efficiently convert glutamate to gamma-aminobutyric acid.

**Results:**

Pyridoxal-5'-phosphate did not affect the cell growth and gamma-aminobutyric acid production of *Lb. brevis *NCL912. Temperature, pH and initial glutamate concentration had significant effects on the cell growth and gamma-aminobutyric acid production of *Lb. brevis *NCL912. The optimal temperature, pH and initial glutamate concentration were 30-35°C, 5.0 and 250-500 mM. In the following fed-batch fermentations, temperature, pH and initial glutamate concentration were fixed as 32°C, 5.0 and 400 mM. 280.70 g (1.5 mol) and 224.56 g (1.2 mol) glutamate were supplemented into the bioreactor at 12 h and 24 h, respectively. Under the selected fermentation conditions, gamma-aminobutyric acid was rapidly produced at the first 36 h and almost not produced after then. The gamma-aminobutyric acid concentration reached 1005.81 ± 47.88 mM, and the residual glucose and glutamate were 15.28 ± 0.51 g L^-1 ^and 134.45 ± 24.22 mM at 48 h.

**Conclusions:**

A simple and effective fed-batch fermentation method was developed for *Lb. brevis *NCL912 to produce gamma-aminobutyric acid. The results reveal that *Lb. brevis *NCL912 exhibits a great application potential in large-scale fermentation for the production of gamma-aminobutyric acid.

## Background

Gamma-aminobutyric acid (GABA) is a non-protein amino acid that is widely distributed in nature from microorganisms to plants and animals [1]. It acts as the major inhibitory neurotransmitter in the mammalian central nervous system. In addition, GABA has hypotensive, tranquilizing and diuretic effects, and can prevent diabetes [2-5]. Also, GABA may improve the concentration of plasma growth hormone and the rate of protein synthesis in the brain [6] and inhibit small airway-derived lung adenocarcinoma [7]. Therefore, GABA has potential as a bioactive component in foods and pharmaceuticals [8]. However, the direction addition of chemical GABA to food is considered unnatural and unsafe [8-10]. So it is necessary to find a natural method to produce and increase GABA in food.

Recent studies have shown that some lactic acid bacteria (LAB) can produce GABA [9,11-19]. LAB possess special physiological activities and are generally regarded as safe (GRAS), and have been extensively utilized in food industries for a long time [20-23]. It is clear that the GABA production by LAB is natural and safe. In addition, the bio-synthetic production of natural GABA produced by LAB for the manufacturing of food can make full use of the health-promoting properties of GABA and LAB themselves. In recent years, the GABA production by using LAB as bacterial cell factories has therefore been a focus of research [8]. Some fermented products enriched in GABA using GABA-producing LAB as starters such as dairy products [2,3,24,25], black raspberry juice [9], soymilk [26], kimchi [10], and cheese [27] have been developed. The GABA-producing ability varies widely among the strains of LAB, and some GABA-producing LAB strains have shown a great promise potential in large-scale fermentation for the production GABA [11,13,15,16,19,28-30].

Since the primary goal of fermentation is the cost-effective and simple production of bio-products, it is important to select a proper process that allows of the highest yielding of the target product. In batch fermentation, substrate should be put in the tank once only. The thing is that the higher initial concentration of fermentation substrate can inhibit the cell growth or waste material resource, and the lower concentration of substrate can not meet the need of high production. Fed-batch culture can make up the weakness and has been widely applied in the production of various bioproducts [31-34]. During fed-batch cultivation, one or more components are supplied to the fermentor while cells and products remain in the tank until the end of operation [31]. A proper initial substrate concentration not inhibiting cell growth can be selected in a fed-batch fermentation, and the limitation component can be added with feeding in the fermentation course. It may help to obtain a high yield and productivity. We previously screened a high GABA-producing *Lb. brevis *NCL912 [11] and the GABA concentration reached 345.83 mM in the optimized fermentation medium [35]. To further increase the yielding of GABA, the effects of pyridoxal-5'-phosphate (PLP), pH, temperature and initial glutamate concentration on the GABA production by *Lactobacillus brevis *NCL912 were firstly determined using flask fermentation in this work. Then a simple process for efficient production of GABA by fed-batch fermentation using *Lb. brevis *NCL912 was developed.

## Results and discussion

### Effect of PLP on GABA production and bacterial growth

**Figure 1:**
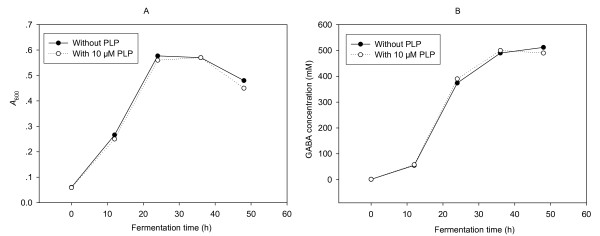
**Influence of PLP on the cell growth (A) and GABA production (B) of *Lb. brevis *NCL912**.

Glutamic acid decarboxylase (GAD, EC 4.1.1.15) catalyzes the irreversible α-decaboxylation of glutamate to produce GABA. GAD uses PLP as coenzyme. Therefore, from the theoretical point of view, an addition of PLP to medium may be a workable method to increase GAD activity to enhance synthetic capacity of GABA. Previous studies had shown that the addition of PLP to medium could effectively increase the GABA production of LAB [15,19]. In our present study, however, the addition of PLP neither affected the cell growth (Figure 1A) nor increased GABA content (Figure 1B) in the fermentation. The possible reason was that NCL912 cells could synthesize sufficient PLP for themselves.

### Effect of temperature on GABA synthesis and bacterial growth

**Figure 2:**
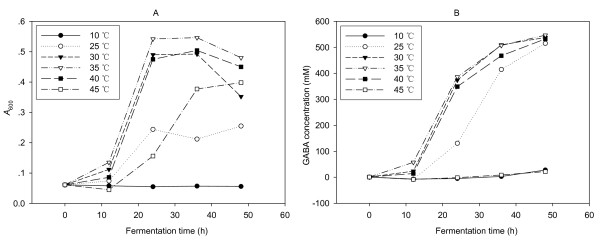
**Influence of fermentation temperature on the cell growth (A) and GABA production (B) of *Lb. brevis *NCL912**.

Figure 2 shows that the considerable variation in the yields of both bacterial growth and GABA production under different fermentation temperatures. The bacterial growth increased with the increase of temperature and peaked at 35°C, then decreased with the increase of temperature. For GABA production, a trend similar to the bacterial growth was observed. It was clear that high cell density was required for effective synthesis of GABA. On the other hand, GABA concentration at 30°C was almost the same to that at 35°C but biomass at 30°C was less than that at 35°C. In addition, NCL912 could not produce GABA at 45°C while the strain could grow under this temperature. These data indicated that appropriate temperature was beneficial to produce GABA, and excessively high temperature was unfavorable to the GABA production. The above results indicated that high efficient conversion glutamate to GABA needed not only high cell density but also appropriate temperature. By comprehensive consideration of the above data, 32°C was selected for the following tests.

### Effect of pH on GABA synthesis and bacterial growth

**Figure 3:**
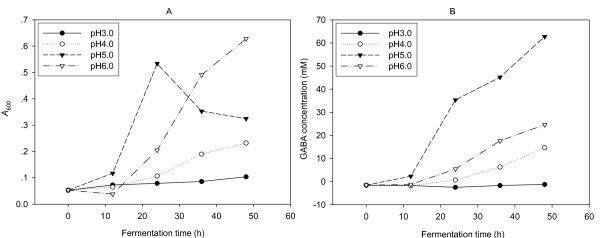
**Influence of pH on the cell growth (A) and GABA production (B) of *Lb. brevis *NCL912**.

It was reported that the GABA biosynthesis in LAB was strictly pH regulated [15,19,36]. To investigate the effect of different pH levels on the production of biomass and GABA of NCL912 in the course of fermentation, the initial pHs of the media were adjusted to 3, 4, 5 and 6, respectively. During the fermentation process, the pHs were adjusted to corresponding initial values immediately after sampling, respectively. As shown in Figure 3, pH has a significant effect on the production of biomass and GABA. The strain could hardly grow at pH3.0 and almost no GABA produced. A highest yield of GABA was obtained at pH5.0 in the fermentation course even if the biomass was less than that of pH6.0 after 24 h. The optimal pH value for the GABA production was 5.0 that accorded with the previous reports about the optimal pH values for maintaining the activity of LAB GADs were in the range of 4.0 to 5.0 [14,37-39]. The higher or lower pH may lead to the partial loss of the GAD activity.

pH 5.0 was also the optimal pH for the cell growth, though the biomass decreased drastically after 24 h. However, the biomass kept increasing during the fermentation course when pH values were maintained at 4.0 or 6.0. It might be the combined inhibitory effect of high concentration of GABA and H_2_SO_4_. For pH5.0, more GABA was produced than pH4.0 and 6.0. The decarboxylation of glutamate to GABA catalyzed by GAD takes the following general form [1,8]:

$$\text{L-glutamate}+{\text{H}}^{+}\overset{\text{GAD}}{\to }\text{GABA}+{\text{CO}}_{2}$$

Decarboxylation of glutamate occurred in LAB results in the stoichiometric release of the end product GABA and the consumption of a proton. The net effect of this reaction is to increase the alkalinity of the cytoplasm and environment. For pH5.0, more H_2_SO_4 _was therefore supplemented into the fermentation broth in order to offset pH increase arising from the decarboxylation.

### Effect of initial glutamate concentration on GABA production and bacterial growth

**Figure 4:**
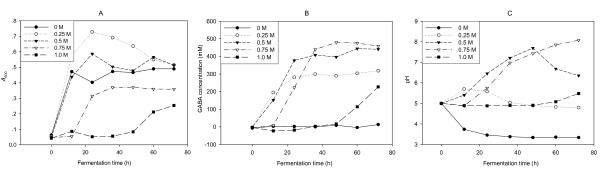
**Effect of initial sodium L-glutamate concentration on the growth (A) and GABA production (B) of *Lb. brevis *NCL912; pH profile (C) of fermentation broths fermented by *Lb. brevis *NCL912 with different initial sodium L-glutamate concentrations**.

As shown in Figure 4A, a moderate addition of glutamate to the medium resulted in an increase in biomass. LAB can metabolize sugars to produce large amount of low weight molecule organic acids, resulting in an acidic environment that was hard for bacteria growth. Some LAB can employ GAD system for maintaining neutral cytoplasmic pH when the external pH drops because the decarboxylation of glutamate within the LAB cell consumes an intracellular proton. In Figure 4C, the pH value of the culture without glutamate decreased to 3.4 after 24 h of fermentation. pH values of the cultures supplemented with glutamate, however, are generally above 5.0. It implied that GAD system of *Lb. brevis *NCL912 acted under low pH and resulted in an increase of pH in medium with glutamate, and protected cell survival from acidic condition. The role of GAD conferring acid resistance to microbial cells was further verified in the present study. On the other hand, the cell growth and biomass decreased with the increase of glutamate concentration at the given levels (0.25, 0.5, 0.75 and 1.0 M). It was apparent that extra high concentration of glutamate was harmful to the strain growth. An addition of 0.25-0.5 M of glutamate was suitable for the strain NCL912 growth and production of GABA.

### Fed-batch production of GABA with pH control

**Figure 5:**
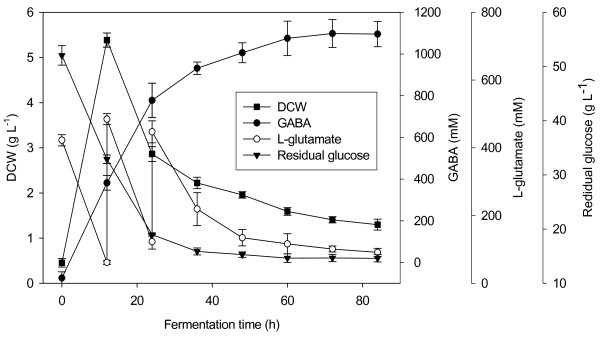
**Time courses of GABA production, residual glutamate, dry cell weight, and residual glucose in fed-batch fermentation**. Data are the means of three independent experiments ± standard deviations (n = 3) analyzed in duplicate.

In the fed-batch process, the initial concentration of glutamate in medium was 400 mM, for the cell growth was strongly inhibited when glutamate exceeded 500 mM. The time courses of GABA production, residual glutamate, DCW, and residual glucose were tested (Figure 5). The cell growth occurred immediately after inoculation and biomass rapidly increased at the first 12 h. And then the biomass dramatically decreased due to the combined inhibitory effect of high concentration of GABA, glutamate and H_2_SO_4_. The concentrations of GABA and glutamate were 381.6 mM and 484.6 mM at 12 h, respectively. In addition, about 100 ml of 10 N H_2_SO_4 _was supplemented into the fermentor in order to offset pH increase arising from the glutamate addition and the decarboxylation. Such harsh conditions were certainly detrimental to the cells and therefore resulted in a sharp decline in biomass after 12 h. The cell growth was almost completely inhibited after 36 h. Without question, compared to feeding strategies maintaining low level glutamate, the current utilized feeding strategy strongly inhibited the cell growth. This feeding strategy, however, was simple and energy-saving. The most important was that the current fermentation method still exhibited powerful capacity of synthesis of GABA (reaching 1095.63 ± 61.03 mM at the end of the fermentations). The biosynthetic kinetics indicate that the GABA concentration increased rapidly with the fermentation time from 0-36 h, increased slowly from 36-60 h, and kept constant after 60 h. The GABA concentrations at 36, 48 and 60 h were 931.50 ± 29.65, 1005.81 ± 47.88 and 1075.05 ± 82.72 mM, respectively, which were obviously higher than that (345.83 mM) [35] before the optimization. Based on a comprehensive consideration of the GABA concentration, energy conservation and fermentation period, 48 h of fermentation was recommended in the future practical production. The volume of the fermentation broth was increased to about 3.75 L due to the inoculation and feed. Residual glutamate and glucose were 134.45 ± 24.22 mM and15.28 ± 0.51 g L^-1 ^at 48 h. Total 738.24 g glutamate (plus the glutamate in the seed medium) was added into the fermentation medium, in which the converted glutamate was 705.81 ± 33.60 g according to the generated GABA mol number, and the residual glutamate was 94.35 ± 17.00 g. This good balance of glutamate added, converted and residual showed that the added glutamate did not participate in other metabolisms. Complete conversion of substrates was beneficial to save material and purify the end product from culture broth. In further practical applications, an addition of 112.28 g glutamate at 24 h, and 35 g L^-1 ^of glucose in the medium are suggested.

## Conclusions

In this study we investigated the effects of PLP, temperature, pH and initial glutamate concentration on the GABA production and bacterial growth of *Lb. brevis *NCL912. PLP did not affect the bacterial growth and the GABA production. In contrast, temperature, pH and initial glutamate concentration had a significant effect on the cell growth and the GABA production, and the conditions were optimized. Then a simple and effective fed-batch fermentation method was developed as follows. The fermentation medium [35] was inoculated with 10% (v/v) seed culture grown to early exponential phase at 32°C. The fed-batch fermentation was carried out under the following conditions: temperature 32°C, agitation speed 100 rpm, pH 5.0, and fermentation time 48 h. 280.70 g and 224.56 g of glutamate were fed into the bioreactor at 12 h and 24 h, respectively. The GABA concentration reached 1005.81 ± 47.88 mM at 48 h. To ensure a complete utilization of glutamate and glucose, 400 mM of glutamate and 35 g L^-1 ^of glucose in the initial medium, and the addition of 280.70 g glutamate at 12 h and 112.28 g glutamate at 24 h into the bioreactor, are suggested.

## Methods

### Strain, medium and cultivation

GABA-producing *Lb. brevis *NCL912 was isolated by our laboratory from paocai, a Chinese traditional fermented vegetable [11]. In a previous study, we optimized the fermentation medium for production of GABA by *Lb. brevis *NCL912, and this medium was theoretically composed of (g L^-1^) [35]: glucose, 55.25; soya peptone, 30.25; MnSO_4_·4H_2_O, 0.0061; and Tween-80 1.38 mL L^-1^. To facilitate the calculation and preparation of the medium in practice, we adjusted the medium components to (g L^-1^): glucose, 50; soya peptone, 25; MnSO_4_·4H_2_O, 0.01; and Tween 80, 2 mL L^-1^. Unless otherwise emphasized, the initial sodium L-glutamate concentration in the medium was 500 mM. The seed medium was composed of (g L^-1^): glucose, 50; soya peptone, 25; MnSO_4_·4H_2_O, 0.01; L-glutamate, 150 mM; and Tween 80, 2 mL L^-1^. Nitrogen sources, glutamate and the other compositions were autoclaved separately at 121°C for 20 min and mixed together prior inoculation. *Lb. brevis *NCL912 was cultured in the seed medium at 32°C for 10 h till the absorbance value at 600 nm (*A*_600_) between 4.0 and 6.0 and then used for seed culture inoculation. Culture condition optimization was conducted in 250-mL Erlenmeyer flasks that contained 100 mL of the medium. The flasks were inoculated with 10% (v/v) seed culture grown to early exponential phase, and incubated under static conditions in an incubator. The fed-batch fermentation was carried out in a 5-L fermentor (Labo-controller MDL-8C; B. E. Marubishi, Tokyo, Japan) under following conditions: medium volume 3 L, inoculum size 10% (v/v), temperature 32°C, pH 5.0, agitation speed 100 rpm, and fermentation time 84 h. The pH was kept constant at 5.0 with addition of 10 N H_2_SO_4_. 280.70 g and 224.56 g glutamate were supplemented into the bioreactor at 12 h and 24 h, respectively. Each flask fermentation was performed in two replicates and the fed-batch fermentation was performed in three replicates.

### Analytic procedures

Glutamate and GABA concentrations in the culture broths were determined by pre-staining paper chromatography [40]. Cell growth was monitored by measuring *A*_600 _on a TU-1901 UV-vis spectrophotometer (Beijing Purkinje General Instrument, China). Dry cell weight (DCW) was calculated by a consistent calibration curve of DCW versus *A*_600_. Glucose concentration was determined by 3, 5-dinitrosalicylic acid method [41]. Each sample was analyzed in duplicate and the mean values were calculated.

## Competing interests

The authors declare that they have no competing interests.

## Authors' contributions

The initiative for this work came from YC. YC and HL designed the experiments; HL and TQ carried out the experimental work; HL and GH analyzed data; YC and HL drafted the manuscript. All authors read and approved the final manuscript.
